# Theoretical understanding of three-dimensional, head-free gaze-shift

**DOI:** 10.1186/1471-2202-15-S1-P184

**Published:** 2014-07-21

**Authors:** Mehdi Daemi, Douglas Crawford

**Affiliations:** 1 Department of Biology, York University, Toronto, ON, Canada; 2 Centre for Vision Research, York University, Toronto, ON, Canada

## 

Shifting the line of sight is naturally accomplished by the movements of both eye and head. We are studying the oclumotor system responsible for planning such head-free gaze-shifts. This includes not only the study of the kinematic mechanisms for coordination of eye and head in three-dimensional space, but also an inquiry into the nature of the internal representations that underlie the observed behavior. The latter is believed to be based on neural representations of sensory signals, coding information is receptors’ frame of reference, being transformed into representations of motor commands, coding information in effectors’ reference frame.

**Figure 1:**
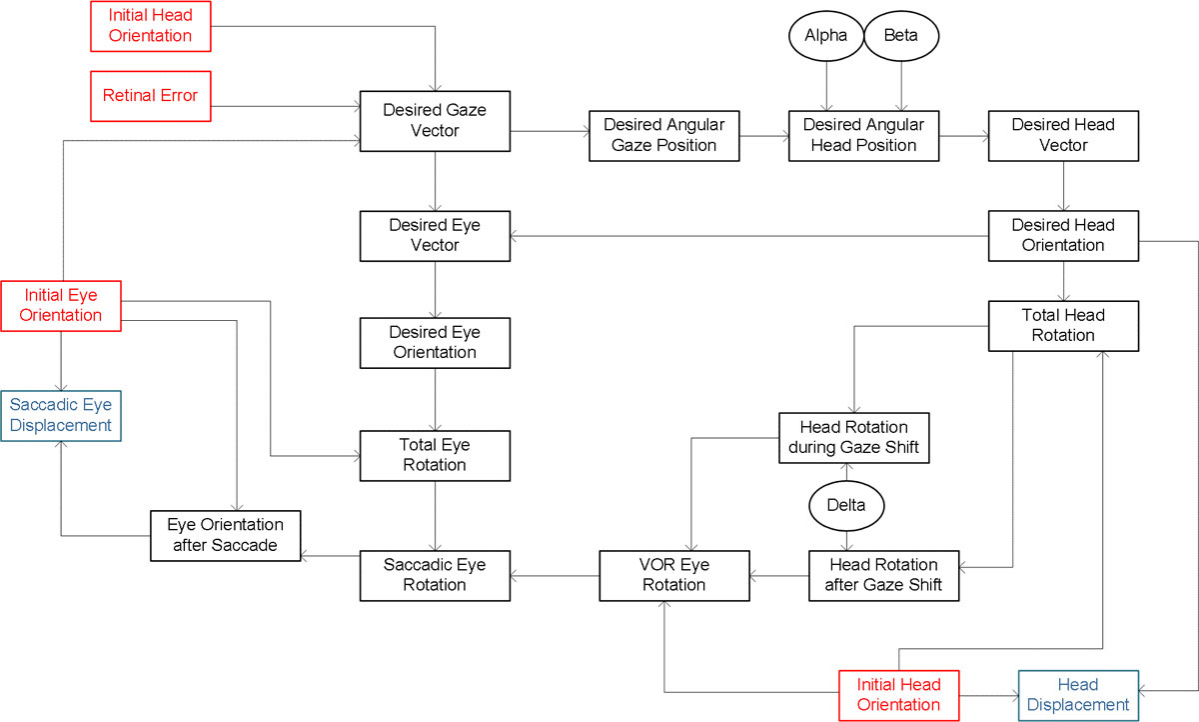
Flow of information in the static kinematic model. Red rectangles show model inputs. Blue rectangles show model outputs. Black ovals are the model parameters.

At the behavioral level, we propose a kinematic model which gets retinal error and initial eye and head orientations as input and describes an experimentally-inspired sequence of rotations including saccadic eye movement, head movement and vestibule-ocular reflex (VOR). Experimentally observed constraints, Listings’ law for eye and Fick strategy for head [1], have been applied. Independent parameters have been defined to control the amount of the head rotation and its contribution to gaze. Figure 1 shows the flow of information in the model in which input and output signals are shown in red and blue boxes respectively and each signal is computed specifically based on the signals from which it receives input.

At the representation level, we have used the neural engineering framework (NEF) [2] to implement a neurophysiologically realistic model of the system. Signals in the kinematic model, shown in figure 1, were considered multidimensional vectors represented by a combination of nonlinear encoding and weighted linear decoding. Computation of each variable from other signals was implemented by transformation of the representations: nonlinear functions of multiple variables characterized as a biased linear decoding of some higher-dimensional representation in a population. We have considered the neurophysiological evidence on the brain areas encoding different signals (e.g. representation of target relative to eye in SC or representation of eye movement relative to head in PPRF and riMLF) as constraints on their representations in our model.

The success of our theoretical study will be evaluated by its success in simulating the known behavior and replicating internal neural signals resembling those recorded by neurophysiologists. The kinematic model has been evaluated based on successfully simulating the known behavior: the accuracy of the gaze shifts and obeying the kinematic constraints for eye and head. The neural network model will be evaluated based on its success in replicating the internal neural signals resembling those recorded by neurophysiologists: the frames of reference and position dependencies of the artificial units.
